# Site-Specific Native Antibody-Conjugated Microbubbles for Molecular Ultrasound Imaging of Hepatocellular Carcinoma

**DOI:** 10.21203/rs.3.rs-8655411/v1

**Published:** 2026-02-13

**Authors:** James Hui, Yonghwan Shin, Janet Pham, Po-Chun Chen, Aravinth Ruppa, Veronica Hankil, Lea Guo, Hanif Saifurrahman Kalamullah, Naoki Kaneko, Jason Chiang

**Affiliations:** UCLA Health; UCLA Health; UCLA Health; UCLA Health; UCLA: University of California Los Angeles; UCLA: University of California Los Angeles; UCLA Health; UCLA: University of California Los Angeles; UCLA Health; UCLA Health

**Keywords:** Contrast-enhanced ultrasound, Light Activated Site-Specific Conjugation, Microbubble, Glypican-3, Hepatocellular carcinoma

## Abstract

**Purpose:**

To develop a microbubble-based contrast agent using light-activated site-specific conjugation (LASIC) for targeted molecular ultrasound imaging of glypican-3 (GPC3) positive hepatocellular carcinoma (HCC).

**Materials and Methods:**

Azide-functionalized microbubbles were conjugated with commercially available anti-GPC3 antibodies using a LASIC DBCO adapter, which enables precise site-specific labeling at the antibody’s heavy chain without compromising antigen-binding affinity. Binding specificity was confirmed through pre-blocking experiments with free anti-GPC3 antibodies in HCC cell lines. Fluorescence microscopy and quantitative image analysis were employed to evaluate cell-binding efficiency. For in vivo validation, conjugated microbubbles were intravenously administered into mice bearing orthotopic HepG2 xenografts, followed by contrast-enhanced ultrasound (CEUS) imaging at 0 seconds, 30 seconds, and 10 minutes post-injection to assess tumor signal enhancement and persistence.

**Results:**

LASIC chemistry enabled covalent and site-specific antibody attachment to microbubbles. In vitro assays demonstrated significantly increased binding of targeted microbubbles to GPC3-positive cells compared to antigen-blocked controls. In vivo CEUS imaging revealed rapid and sustained tumor-specific signal enhancement within 30 seconds of injection, persisting up to 10 minutes. In contrast, control microbubbles showed diminished signal intensity post-injection.

**Conclusion:**

LASIC-conjugated anti-GPC3 microbubbles exhibit efficient, specific, and durable binding to GPC3-expressing HCC cells both in vitro and in vivo. This strategy enables real-time, image-guided molecular profiling via CEUS and holds promise for precision imaging and theranostic applications in liver cancer management.

## Summary Statement

This study introduces site-specifically labeled microbubbles for targeted CEUS imaging of HCC, thereby enables noninvasive molecular profiling using off-the-shelf antibodies and offers a clinically translatable strategy for personalized liver cancer diagnostics.

## Introduction

Hepatocellular carcinoma (HCC) is the fourth-leading cause of all cancer-related deaths worldwide, accounting for approximately 90% of primary liver cancers (1). While the standard of care diagnosis of early-stage HCC traditionally relies on contrast-enhanced CT or MRI (2), these modalities suffer from limitations including lack of real-time visualization, limited outpatient imaging availability, ionizing radiation (with CT), and motion artifact (with MRI). Contrast-enhanced ultrasound (CEUS) offers key advantages in facilitating HCC detection, including real-time evaluation, absence of ionizing radiation, and favorable safety profiles of microbubble contrast agents (3–5).

Despite its proven utility in diagnosing HCC, CEUS does not provide information about the underlying tumor molecular characteristics, which may be leveraged for prognostic purposes and determine optimal treatment strategies(6). While needle biopsies can be performed to obtain tissue in cases of suspected HCC, they are usually reserved for LR-4 or LR-M lesions, are prone to sampling heterogeneity and pose procedure-related risks(7). These limitations highlight the need for reliable ways to detect tumor markers in HCC.

Given CEUS’ favorable safety profile and ready accessibility, a compelling approach to non-invasively characterize cancer surface markers is to conjugate an antibody of interest onto contrast ultrasound microbubbles. Microbubbles that are currently being used clinically in CEUS are nontargeted, limiting them to only imaging perfusion and unable to provide molecular-level information. While there have been various precedents in developing targeted microbubbles, the techniques used for antibody attachment are often inefficient and tedious, thereby restricting their wider adoption beyond proof of principle demonstrations. For example, the most commonly used amine modification chemistry results in the antibodies that are often mis-oriented on the microbubble and indiscriminately linked via any number of lysine residues, leading to heterogeneous products with variable pharmacokinetics, toxicity and affinity for antigen(8). Another class of widely used conjugation technique - based on biotin-avidin/streptavidin binding, not only suffers from similar shortfalls since biotinylation also occurs indiscriminately on lysine groups, but what’s more is also poorly suited for clinical uses due to its immunogenicity and interference from endogenous biotins. Consequently, considerable efforts have been made to develop site-specific conjugates for a variety of antibody-based products– with the goal of achieving more precise control of the modification sites and higher production efficiency(9).

Light Activated SIte-Specific Conjugation (LASIC) is a previously developed site-specific antibodylabeling technique of native antibodies via photochemistry(10). This technique facilitates the formation of a single covalent bond between a 60mer peptide adapter containing the desired conjugate and each Fc region on IgG when exposed to gentle long wavelength UV light, leading to more homogeneous, sitespecific attachments. As this technique preserves antibody function and orientation, can be used with any off-the-shelf antibodies, and can be readily combined with the robust azide based click chemistry, it maybe well-suited for efficiently attach desired antibodies onto the fragile microbubbles. Glypican-3 (GPC3) is well recognized as a highly specific surface marker of HCC, carries prognostic values, and is the molecular target for several therapies in development (11–15). By developing targeted microbubbles against GPC3 in this study, we hope to demonstrate the efficient production of targeted, site-specifically labeled microbubbles for molecular imaging using CEUS.

## Materials and Methods

### Cell Culture

Human hepatocellular carcinoma (HCC) cell lines—SNU423, SNU449, and HepG2—were obtained from the American Type Culture Collection (ATCC, Manassas, VA, USA). SNU423 and SNU449 cells were cultured in either RPMI 1640 or Dulbecco’s Modified Eagle Medium (DMEM), both supplemented with 10% fetal bovine serum (FBS), 100 U/mL penicillin, and 100 μg/mL streptomycin. All cell lines were maintained at 37 °C in a humidified incubator with 95% air and 5% CO_2_.

### Conjugation of the GPC3 Antibody with microbubbles

Native (Cat# BE0404, Bio X cell, Lebanon, NH, USA) and FITC-labeled Anti-GPC3 antibodies (Cat# NBP3–28387F, Novus Biologicals, Centennial, CO, USA) were site-specifically modified using oYo-link DBCO (AlphaThera, Philadelphia, PA, USA) according to the manufacturer’s instructions, specifically 20 μg of anti-GPC3 antibody was incubated with oYo-link DBCO and exposed to long wavelength (365 nm) UV light on ice for 2 hours to enable photocrosslinking. The conjugated antibody was then purified using oYo-capture magnetic beads and washed three times with PBS. Usphere^™^ labeler microbubbles containing azide-modified phospholipids (Scintica, Webster, TX, USA), which are based on a commercially available preclinical contrast agent with established in vivo dosing and imaging safety guidelines provided by the manufacturer, were agitated with the UltraMix^™^ device for 40 seconds at room temperature to generate a homogeneous suspension with a particle concentration of 1–5 × 10^10^ microbubbles/mL, according to the manufacture’s instruction. The purified oYo-link DBCO conjugated antibody was mixed with 20 μl of azide functionalized microbubbles at room temperature for at least 2 hours to allow the click chemistry covalent bond to form, resulting in stable antibody microbubble conjugates.

### Dynamic Light Scattering, Zeta Potential, and Stability Measurements

Dynamic light scattering (DLS) and zeta potential measurements were performed on the Zetasizer Nano Zs analyzer (Malvern Panalytical, Malvern, UK) to characterize microbubble size distribution and surface charge. For both measurements, samples were diluted 1:10 in deionized water. Zeta potential readings were obtained using U-shaped cuvettes, while DLS measurements utilized plastic cuvettes. All analyses were performed at 25 °C and repeated in triplicate. Data were processed using Zetasizer software. Microbubble stability was evaluated by monitoring the number of intact microbubbles over time. Microbubbles were diluted 1:50,000 (v/v) in culture medium and incubated at 37 °C. At the indicated time points from 0 to 90 min, microbubble counts were quantified by light microscopy.

### Western Blot Analysis

The harvested SNU423, SNU449, and HepG2 cells were washed with phosphate buffered saline (PBS) and dissolved in 20 mM Tris-HCl, pH7.5, lysis buffer. The protein samples were separated using 4–12% SDS-PAGE and transferred to a 0.2 μm nitrocellulose membrane. After blocking with 5% non-fat dry milk (Bio-Rad, Irvine, CA, USA), the membrane was incubated overnight at 4 °C with a primary antibody and washed with Tris-buffered saline with Tween 20 (TBST). The membrane was washed with TBST, incubated at room temperature with a secondary antibody for 1 h, and visualized using an ECL reagent (Thermo Fisher Scientific, San Diego, CA, USA). All experiments were performed in triplicate. Antibodies used in this study are follow: GPC3 antibody (1:5000) from Bio X Cell (Lebanon, NH, USA); β-Tubulin antibody (1:5000) from Abcam (Cambridge, MA, USA); HRP-conjugated anti-mouse antibody (1:5000) from Abcam (Cambridge, MA, USA).

### Immunofluorescence

HepG2 cells were grown on cell culture slides (Thermo Fisher Scientific, San Diego, CA, USA) and treated with oYo-link DBCO-anti-GPC3 conjugated Microbubbles for 30 min. Then, these cells were stained with DAPI (Thermo Fisher Scientific, San Diego, CA, USA). The cells were washed with PBS and visualized by a Nikon Confocal Laser Scanning Confocal microscope.

### PDMS Phantom Vessel Model

PDMS (Sylgard 184, 10:1 w/w base to curing agent) was cast into a rectangular acrylonitrile-butadienestyrene (ABS) mold featuring a central 3.15-mm-diameter cylindrical core, creating a phantom with vessel-like cylindrical lumen at its center. To enhance hydrophilization, the PDMS model was soaked in 10% (3-aminopropyl) trimethoxysilane (APTMS) in 100% ethanol overnight and then washed with water. The luminal surface of the model was then activated with sulfo-SANPAH followed by UV irradiation and was subsequently treated with fibronectin (40 μg/mL). HepG2 cells were cultured in DMEM supplemented with 10% FBS and 100 U/ml penicillin + 100 μg/ml streptomycin at 37 °C in a CO_2_ incubator (95% air, 5% CO_2_) then seeded into the PDMS phantom vessel model. To promote a uniform cell adhesion on the vessel luminal surface, the model was seeded in a 3D rotating instrument at 1 rotation per minute placed in the CO2 incubator. After establishing the PDMS cell culture model, oYo-link anti-GPC3 antibody-conjugated microbubbles were injected into the vessel using a 1 mL syringe, incubated to allow binding to HePG2 cells, and visualized by a Nikon Confocal Laser Scanning Confocal microscope.

### HepG2 Cells-Derived Orthotopic Implantation and Ultrasound Imaging

HepG2 cells were suspended in RPMI containing 25% Matrigel (356234, Life Sciences) such that 100 μL volume contained 1×10^6^ cells to inject subcutaneously into mouse flanks. After three weeks, tumors were excised from the flanks and orthotopically implanted into the liver following a standard protocol(16). Briefly, 7-week-old nude mice (homozygous for Foxn1<nu>, male, Jackson Laboratories) were anesthetized using isoflurane, and the liver was exposed. A 70 mg tumor was attached to the left-lateral lobe of the liver using 5.0 VICRYL suture (NC2872069, Fisher Scientific, Waltham, MA, USA) and wrapped with a 1×0.5 cm piece of SURGICEL further protect the attachment and minimize bleeding. Incisions were closed using wound clips, and mice were set up for recovery with a post-surgery care protocol. After four weeks of implantation, the mice were injected into the tail vein with microbubbles or with oYo-Link DBCO-anti-GPC3 conjugated microbubbles and ultrasound imaging was performed using a linear array transducer (el 18–4). Contrast-mode imaging was conducted at a low mechanical index (MI = 0.06) to preserve microbubble integrity, with a frame rate of 23Hz. The imaging depth was set to 3.5 cm, and gain settings were kept constant across experiments. Dual-mode imaging was used to simultaneously acquire contrast-enhanced and B-mode images. The injected microbubble dose was selected based on the manufacturer’s protocol for tumor imaging in mice. All animal protocols in this study were approved by the Division of Laboratory Animal Medicine (DLAM) at the university.

## Results

### LASIC-based Conjugation and Characterization of GPC3 Targeted Microbubble

**Figures 1:**
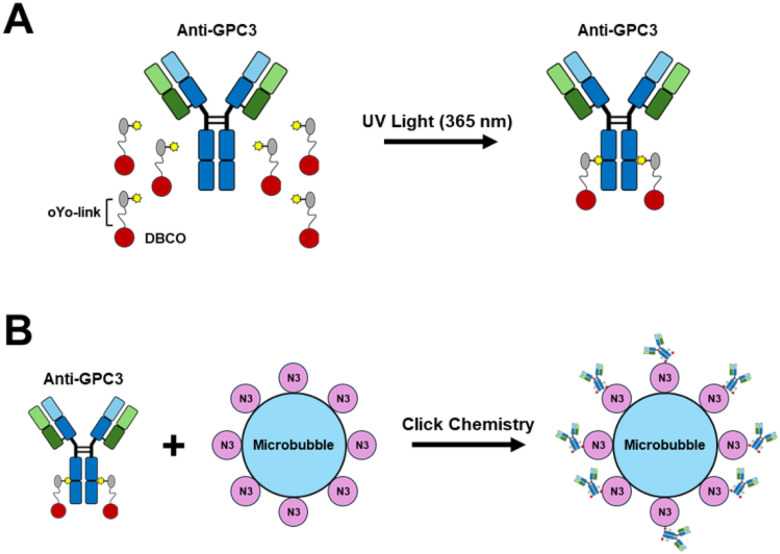
Schematic representation of LASIC-based conjugation of anti-GPC3 antibody to microbubbles. **(A)** LASIC utilizes gentle long wavelength UV light (365 nm) to covalently conjugate a small, 6kD peptide adapter (oYo-link DBCO) onto the Fc regions of the antibody, enabling site-specific labeling of the heavy chain by photocrosslinking. **(B)** The oYo-link DBCO conjugated anti-GPC3 antibody were coupled to azide functionalized microbubbles through click chemistry, enabling stable conjugation of oYo link DBCO conjugated anti-GPC3 antibody to the microbubbles surface.

LASIC has previously been introduced as a versatile method to achieve site-specific antibody modification without compromising antigen binding(10). Building upon this method, we developed glypican-3 (GPC3)-targeted microbubbles using an optimized LASIC-based strategy. Specifically, long-wavelength UV light was employed to covalently attach a small peptide adapter containing a Clickchemistry compatible strained octyne moiety (oYo-link DBCO) to the Fc region of a commercially available, “off the shelf” anti-GPC3 monoclonal IgG1 antibody. This site-specific conjugation selectively modified the antibody’s heavy chains while preserving antigen recognition. Following modification, the anti-GPC3 antibodies were conjugated to commercially available azide-functionalized microbubbles via Click chemistry, resulting in stable and uniform antibody attachment to the microbubble surface (Fig. 1).

**Figure 2:**
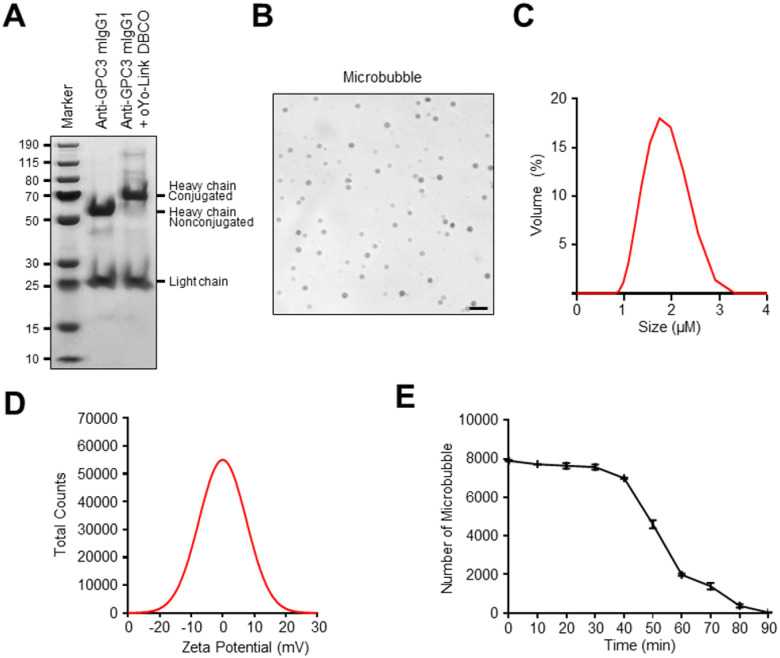
LASIC-based anti-GPC3 conjugation and characterization of microbubbles. **(A)** Reducing SDS-PAGE confirmed site-specific conjugation of human anti-GPC3 mIgG1 heavy chain before and after photocrosslinking with oYo-link DBCO. **(B)** Light microscopy showed azide-containing microbubbles. Scale bars correspond to 10 μm. **(C)** Microbubbles size distribution was measured by dynamic light scattering showing an uniform distribution. **(D)** The zeta potential of the microbubbles was analyzed to assess surface charge distribution. **(E)**The microbubbles were seen to be stable for at least 30 mins at 37 °C.

When the conjugated antibody is examined on SDS-PAGE gel, a shift in the apparent molecular weight of the heavy chain before and after photo-crosslinking with oYo-link DBCO confirmed successful site-specific labeling of antibody (Fig. 2A). Light microscopy visualized the microbubble morphology (Fig. 2B), while dynamic light scattering (DLS) analysis demonstrated consistent size distribution (Fig. 2C). Zeta potential measurements revealed a neutral surface charge, indicative of microbubble stability (Fig. 2D). Additionally, microbubble stability was assessed at 37°C in culture medium, where most microbubbles degraded within 30 minutes, on par with the in vivo stability of clinically used bubbles. (Fig. 2E), indicating reasonable stability in post-reconstitution.

### Binding of GPC3 Targeted Microbubbles to HCC Cells

**Figure 3:**
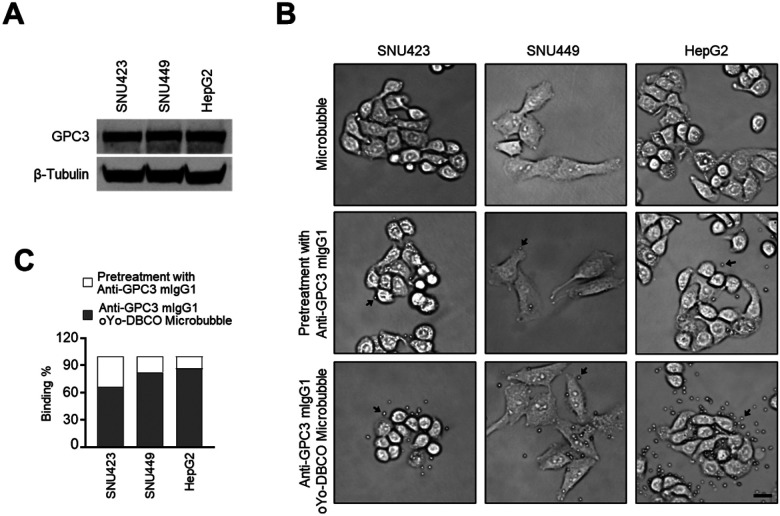
In vitro binding specificity of anti-GPC3 conjugated microbubbles. **(A)** Western blot analysis of whole-cell lysates from SNU423, SNU449, and HepG2 cells using anti-GPC3 antibody, with β-Tubulin as a loading control. **(B)** Light microscopy images showing microbubble binding under three conditions: unlabeled microbubbles (top), oYo-Link DBCO–anti-GPC3 conjugated microbubbles after pre-blocking with free anti-GPC3 antibody (middle), and oYo-Link DBCO–anti-GPC3 conjugated microbubbles (bottom). Scale bars correspond to 50 μm. **(C)** Quantitative analysis of cell-binding efficiency from panel B confirms specific binding of conjugated microbubbles to GPC3-positive cells.

GPC3 is a well-established biomarker and therapeutic target in HCC (11–15, 17, 18). To validate a GPC3-targeted molecular imaging approach, we first confirmed GPC3 expression in HCC cell lines—SNU423, SNU449, and HepG2—using Western blot analysis. All three cell lines exhibited strong GPC3 expression (Fig. 3A).

Next, we evaluated the binding efficiency of microbubbles conjugated with oYo-link DBCO-anti-GPC3 antibodies. Cells were incubated under three conditions: with unconjugated microbubbles, with oYo-link DBCO-anti-GPC3-conjugated microbubbles, and with conjugated microbubbles following pretreatment with free anti-GPC3 antibodies to block surface epitopes. Bright-field microscopy revealed robust binding of the conjugated microbubbles to HCC cells, while pre-blocking with free antibodies significantly reduced binding (Fig. 3B). Quantitative analysis confirmed these observations (Fig. 3C).

**Figure 4:**
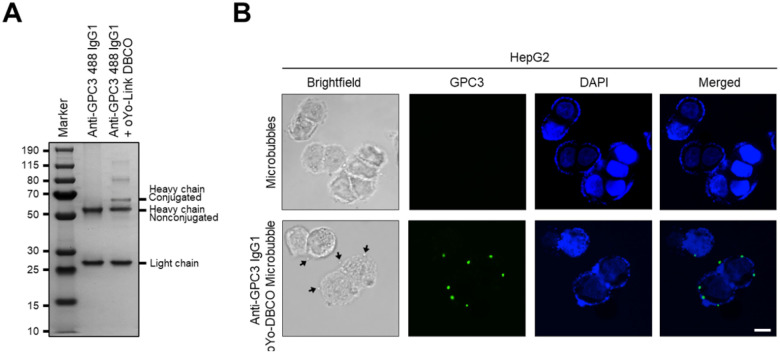
Cellular targeting of fluorescence labeled anti-GPC3 conjugated microbubbles. **(A)** Reducing SDS-PAGE confirmed site-specific conjugation of FITC–labeled anti-GPC3 IgG1 heavy chain before and after photocrosslinking with oYo-link DBCO. **(B)** HepG2 cells were incubated with oYo-link DBCO-FITC anti-GPC3 IgG conjugated microbubbles. Microscopy demonstrated binding of FITC-IgG containing fluorescent microbubbles onto cells. Nuclei were counterstained with DAPI (blue). Representative images of stained cells are shown. Scale bars correspond to 15 μm.

To allow fluorescent assessment of microbubbles binding in models, such as the PDMS vessel model further described below, that have suboptimal bright light penetration, we used a commercial, FITC-labeled anti-GPC3 antibody to conjugate onto azide microbubbles. SDS-PAGE confirmed site-specific photo-crosslinking of oYo-link DBCO to the heavy chain of FITC-labeled anti-GPC3 IgG1 (Fig. 4A). HepG2 cells were treated with the resulting fluorescent anti-GPC3 microbubbles and analyzed via fluorescent microscopy. Strong FITC signals were seen on the cell surface in treated cells, colocalizing with microbubbles seen using bright-field microscopy, (Fig. 4B).

### GPC3 Targeted Microbubble Binding in PDMS Vessel Model

**Figure 5:**
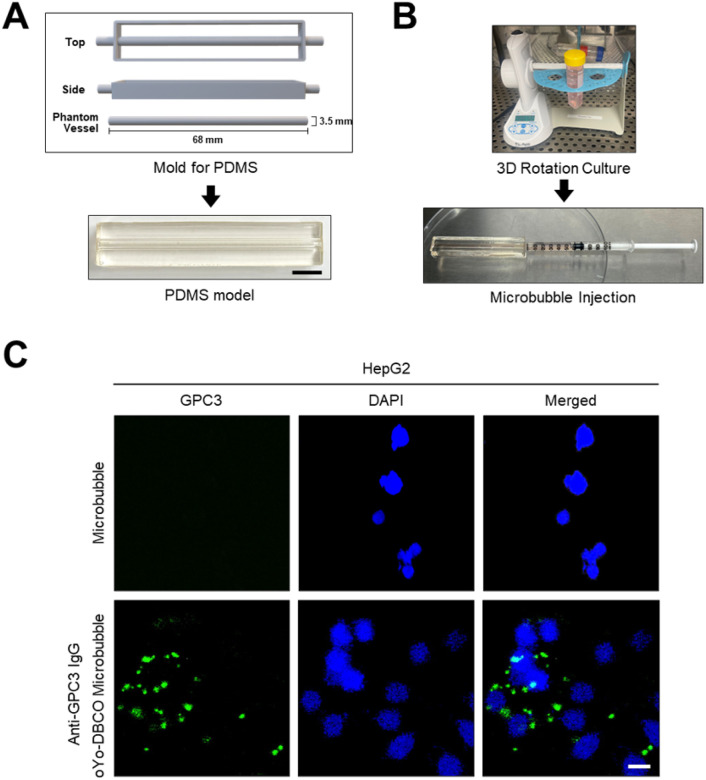
Assessment of targeted fluorescence labeled anti-GPC3-conjugated microbubbles binding in PDMS model. **(A)** Schematic and photograph of the PDMS phantom vessel model used for microbubble flow experiment. Scale bars correspond to 1 cm. **(B)** HepG2 cells were seeded in PDMS models, cultured under a 3D rotation system and subjected to FITC labeled anti-GPC3 conjugated microbubbles injection into the PDMS phantom vessel model. **(C)** HepG2 cells incubated with microbubbles only (upper) or oYo-link DBCO-FITC anti-GPC3 IgG conjugated microbubbles (lower) showed GPC3 specific fluorescence (Green) localized to cell membranes. Nuclei were counterstained with DAPI (blue). Scale bars correspond to 50 μm.

To assess the targeting capability of oYo-link DBCO-anti-GPC3-conjugated microbubbles under vessellike conditions, we developed a polydimethylsiloxane (PDMS) phantom vessel model, adapted from a previously described method(19). The model featured a central cylindrical lumen, which was hydrophilized, coated with human fibronectin, and seeded with HepG2 cells. A 3D rotational culture was applied for 48 hours to promote uniform cell adhesion along the inner surface, thereby simulating a biologic vessel environment (Fig. 5A, 5B).

Following model establishment, FITC anti-GPC3-conjugated microbubbles described in the previous section were introduced via syringe injection and incubated to facilitate binding to HepG2 cells (Fig. 5B). Fluorescence imaging revealed strong FITC signals localized to the cell surfaces in samples treated with the conjugated microbubbles, confirming its ability to bind to GPC3-expressing HCC cells within a vessel-mimicking environment (Fig. 5C).

### Ultrasound Imaging of Orthotopic HCC Xenograft with targeted Microbubbles

**Figure 6:**
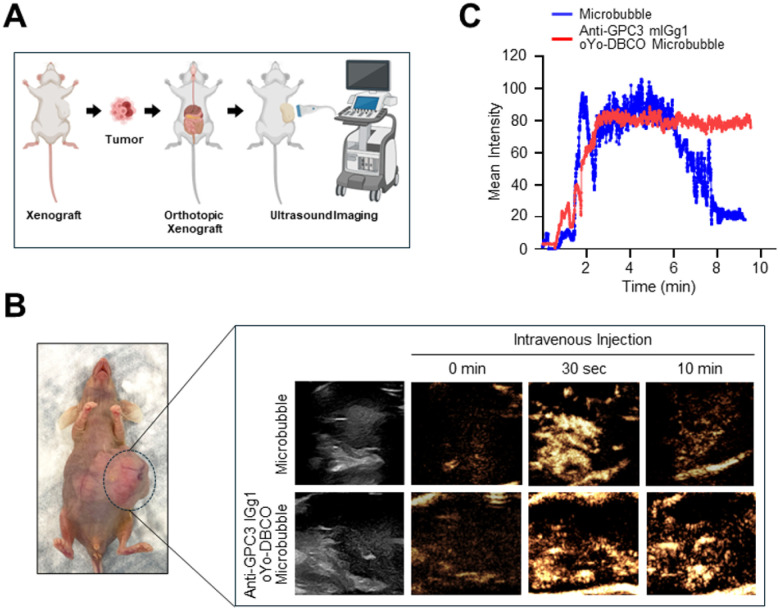
In vivo contrast-enhanced ultrasound of orthotopic HepG2 xenograft with targeted anti-GPC3 conjugated microbubbles. **(A)** Schematic diagram of the experimental workflow showing orthotopic HepG2 xenograft implantation followed by ultrasound imaging after intravenous microbubble injection. **(B)** Mice with orthotopic HepG2 xenograft tumors were injected with control microbubbles (upper) or oYo-Link DBCO-anti-GPC3 mIgG1 conjugated microbubbles (lower) and subsequently imaged by ultrasound. **(C)** Quantitative analysis of signal intensity was performed in the targeted anti-GPC3 conjugated microbubbles group and compared with control.

To assess the tumor-targeting ability of oYo-link DBCO-anti-GPC3 conjugated microbubbles in a physiologically relevant setting, an orthotopic HepG2 xenograft mouse model was established as previously described(16). After tumors developed in the liver, mice received intravenous injections of either unconjugated microbubbles or GPC3-targeted microbubbles, followed by CEUS imaging (Fig. 6A, 6B). CEUS revealed rapid and strong signal enhancement in tumors within 30 seconds of injecting GPC3-targeted microbubbles, persisting for up to 10 minutes (Fig. 6B). In contrast, mice injected with unconjugated microbubbles exhibited perfusion driven enhancement that diminished within 10 minutes. Quantitative analysis confirmed significantly higher and sustained signal intensity in the targeted group compared to controls (Fig. 6C). These findings demonstrate that oYo-link DBCO-anti-GPC3 conjugated microbubbles enable specific and durable imaging of GPC3-positive HCC xenografts, supporting their potential as targeted molecular ultrasound contrast agents.

## Discussion

This study demonstrates the potential of LASIC—a novel site-specific conjugation technique—for advancing ultrasound imaging from purely structural assessment to molecular-level diagnostics. By targeting GPC3, a well-validated surface marker of HCC, with commercially available antibodies, LASIC-labeled microbubbles successfully detected GPC3 expression in cells and in an orthotopic HCC mouse model. This approach enables noninvasive, real-time, and widely accessible evaluation of tumor surface markers, paving the way for integrating molecular phenotyping into routine diagnostic workflows and supporting more personalized management strategies for HCC.

Although previous studies have demonstrated the feasibility of targeted microbubbles, their construction have largely relied on conventional conjugation chemistries that produce heterogeneous and often immunogenic products at low yields—making them difficult to replicate, scale, and translate clinically. These traditional methods, such as non-specific acylation of lysine residues or alkylation of cysteine thiols, have remained largely unchanged for decades and frequently result in promiscuous antibody attachment, compromising product uniformity and function(20). Recent efforts to achieve site-specific conjugation have involved complex antibody engineering—introducing ultra-reactive amines, unnatural amino acids, or peptide tags designed for bioorthogonal reactions(9, 21). While these approaches offer improved specificity, they are labor-intensive, may disrupt microbubble integrity, and require extensive customization for each target antigen(10, 22–24). The LASIC conjugation method produces homogeneous products while preserving the Fab region for antigen binding. Furthermore, by leveraging bioorthogonal click chemistry, the method used in this work maintains the structural integrity of both antibodies and microbubbles, making it ideally suited for molecular imaging applications(10, 25).

CEUS microbubbles possess favorable pharmacokinetic and safety profiles, contributing to their widespread clinical adoption in Europe and FDA approval in the United States (27, 28). The successful application of LASIC-based labeling to microbubbles presents exciting opportunities for molecular profiling across a range of solid tumors. The flexible, plug-and-play conjugation strategy described here enables the use of off-the-shelf antibodies to readily target other potential biomarkers such as HER2 in breast cancer, EGFR in lung cancer, and PSMA in prostate cancer (29, 30). Despite the advantages of ultrasound imaging—including real-time capability and safety—certain limitations remain. These include restricted soundwave penetration through bone or air-filled organs, reduced resolution in deeper tissues, and reliance on operator expertise for image acquisition. However, ongoing advancements in ultrasound technology, such as high-frequency transducers and 3D imaging, may help overcome these challenges. Finally, while LASIC-labeled microbubbles are expected to exhibit minimal immunogenicity—especially compared to biotin/avidin-based systems—further validation in animal models is necessary to confirm their safety and suitability for repeated clinical use.

This study demonstrates the potential of LASIC for efficiently labeling ultrasound microbubbles with antibodies targeting GPC3, a clinically significant surface marker in HCC. By enabling molecular-level imaging through targeted CEUS, this technique offers a powerful, noninvasive method to characterize tumor biology in real time. The anticipated improvements in specificity, stability, and imaging performance of LASIC-conjugated microbubbles may not only enhance diagnostic accuracy for HCC but also support personalized treatment planning across a range of solid tumors. Further preclinical validation and eventual clinical translation will be essential to fully realize the impact of this targeted imaging platform in oncologic radiology.

## Data Availability

Data generated or analyzed during the study are available from the corresponding author by request.

## Key Points:

1. **LASIC enables precise and reproducible antibody conjugation to microbubbles**, overcoming limitations of traditional labeling methods by preserving antigen-binding affinity and ensuring homogeneous product formation.
2. **GPC3-targeted microbubbles demonstrated robust and sustained tumor-specific imaging** in both in vitro and orthotopic HCC mouse models, validating their potential for noninvasive molecular profiling using contrast-enhanced ultrasound (CEUS).
3. **The modularity of the conjugation technique supports broad clinical applicability**, allowing plug-andplay targeting of diverse tumor biomarkers with off-the-shelf antibodies, paving the way for personalized imaging strategies across multiple solid tumors.
